# Association Study of Candidate Gene Polymorphisms with Amnestic Mild Cognitive Impairment in a Chinese Population

**DOI:** 10.1371/journal.pone.0041198

**Published:** 2012-07-20

**Authors:** Xiaoyan Liu, Chunxian Yue, Zhi Xu, Hao Shu, Mengjia Pu, Hui Yu, Yongmei Shi, Liying Zhuang, Xiaohui Xu, Zhijun Zhang

**Affiliations:** 1 School of Clinical Medicine, Southeast University, Nanjing, China; 2 Department of Neurology, Affiliated ZhongDa Hospital of Southeast University, Nanjing, China; University of Ulster, United Kingdom

## Abstract

To investigate the relationship between amnestic mild cognitive impairment (aMCI) and candidate gene polymorphisms in a Chinese population, 116 aMCI patients and 93 normal controls were recruited. Multi-dimensional neuropsychological tests were used to extensively assess the cognitive functions of the subjects. MassARRAY and iPLEX systems were used to measure candidate single nucleotide polymorohisms (SNPs) and analyse allelic, genotypic or haplotypic distributions. The scores of the neuropsychological tests were significantly lower for the aMCI patients than for the normal controls. The distributions of SNPs relating to the amyloid cascade hypothesis (TOMM40 rs157581 G and TOMM40 rs2075650 G), to the cholesterol metabolism hypothesis (ApoE rs429358 C, LDLR rs11668477 G and CH25H rs7091822 T and PLAU rs2227564 CT) and to the tau hypothesis (MAPT/STH rs242562 GG) in aMCI were significantly different than those in normal controls. Interactions were also found in aMCI amongst SNPs in LDLR rs11668477, PLAU rs2227564, and TOMM40 rs157581, between SNPs in TOMM40 rs157580 and BACE2 rs9975138. The study suggests that aMCI is characterised by memory impairment and associated with SNPs in three systems relating to the pathogenesis of AD-those of the amyloid cascade, tau and cholesterol metabolism pathways. Interactions were also observed between genes in the amyloid pathway and between the amyloid and cholesterol pathways.

## Introduction

Mild cognitive impairment (MCI) is an intermediate state between normal aging and dementia in which the cognitive decline is greater than that expected for an individual's age and education level but does not interfere significantly with the activities of daily life. It can be divided into two subtypes: non-amnestic MCI (naMCI) and amnestic MCI (aMCI). aMCI is characterised by memory complaints and deficits and has a high probability of progressing to Alzheimer's disease (AD) at a rate of 10–15% per year [1], [2].

The genetics of late-onset AD (LOAD) has been comprehensively studied. Many studies consistently demonstrated apolipoprotein E (ApoE) ε4 to be a genetic risk factor but does not inevitably result in LOAD. Moreover, there have been 1395 association studies conducted on 695 AD candidate genes, and there are 2973 polymorphisms studied to date according to the Alzgene website (update on 2011-04-18). Candidate gene studies are mainly focused on functional gene polymorphisms relating to AD-physiopathological hypotheses. The discovery of a link between memory loss and the basal forebrain cholinergic deficits in AD patients and the concept of the “cholinergic hypothesis” have triggered many molecular genetic analyses intended to uncover novel risk factors for AD, including enzymes involved in acetylcholine metabolism [3]. Two other hypotheses amongst the most common and persuasive are the amyloid cascade hypothesis and the tau hypothesis. According to the amyloid cascade hypothesis, β-amyloid (Aβ) is generated from the amyloid precursor protein by the sequential actions of β-secretase and γ-secretase, and the imbalance between Aβ production and clearance is a central event in AD that can induce the deposition of Aβ42 oligomers as diffuse plaques, inhibit hippocampal long-term potentiation and impair synaptic function [4]. In the tau hypothesis, the tau protein functions to improve microtubule assembly and stability, but it is changed in AD in two ways, either through phosphorylation or conformational changes that can be induced by the regulation of particular protein kinases and protein phosphatases [5]. Finally, the cholesterol metabolism hypothesis is a major addition to the amyloid cascade hypothesis and the tau hypothesis because cholesterol is a vital component of neuronal membranes, and many processes in the pathogenesis of AD involve membranes [6]. The level of cholesterol is regulated through synthesis, storage, transport, and degradation, and the major players in its metabolism include cholesterol itself, the enzyme β-hydroxy-β-mathylglutaryl-CoA reductase, the cholesterol transport protein ApoE, the adenosine triphosphate binding cassette transporter proteins A1 and G1, low density lipoprotein-related protein (LRP), low density lipoprotein receptor (LDLR), and the oxysterols 24S-hydroxycholesterol and 27-hydroxycholesterol to which cholesterol is converted in the brain and body, respectively [6]. As a result of these hypotheses, candidate gene studies for AD have been widely conducted, resulting in the discovery of many novel susceptibility factors for AD other than ApoE ε4, and the discovery that there are interactions between different genes for AD. For instance, the K-variant of butyrylcholinesterase was demonstrated to modify the risk of LOAD in ApoE ε4 carriers [7]. However, the results from association studies including 32 genome-wide association studies in AD (according to the Alzgene website) were inconsistent. The discrepancy could be explained by several reasons such as ethnic or environmental differences, sample size, and disease severity.

To summarise, although several hypotheses have been well established to date, such as the cholinergic hypothesis, the amyloid cascade hypothesis, the tau hypothesis and the cholesterol metabolism hypothesis, none of these hypotheses has fully accounted for the diversity of the initial events that result in the deposition of senile plaques and neurofibrillary tangles. An increasing number of studies point out that cholesterol is involved in Aβ generation [8]. Several experimental results indicate that Aβ accumulation precedes and drives tau aggregation [9], and Aβ-induced neurotoxicity requires tau [10]. Damaged tissue from Aβ aggregates can activate microglia and enhance the expression of inflammatory factors that have an effect on cholinergic neurons and stimulate astrocytes that eventually amplify proinflammatory signals to induce neurotoxic effects [11]. Therefore in this study, we aimed to investigate the relationship between aMCI and candidate gene polymorphisms in a Chinese population by reporting single gene analyses in the four main pathways related to the pathogenesis of aMCI and AD and investigating the interactions between SNPs in these various genes.

## Materials and Methods

### Subjects and clinical assessments

The diagnosis of individuals with aMCI was performed essentially following Petersen's recommendations [1]: (1) memory complaint, (2) an objective memory impairment on a neuropsychological evaluation: 20 minute delayed recall of auditory verbal learning test (AVLT) score ≤4 for ≥8 years of education, (3) a normal general cognitive function: a Mini-Mental State Examination (MMSE) score ≥24, (4) a Clinical Dementia Rating (CDR) of 0.5 with a rating of at least 0.5 in the memory domain, (5) a normal or only slightly impaired activities of daily living (ADL) score ≤22, and (6) not demented: not sufficient to meet the National Institute of Neurological and Communicative Disorders and Stroke/Alzheimer's Disease and Related Disorders Association criteria for AD. All controls were required to have a CDR of 0, an MMSE score ≥26, and a delayed recall of AVLT score >4 for ≥8 years of education. Participants were excluded from the present study if they had a past history of known stroke (modified Hachinski score ≥4), alcoholism, head injury, Parkinson's disease, epilepsy, major depression or other neurological or psychiatric illness, major medical illness (e.g., cancer, anaemia, thyroid dysfunction), or severe visual or hearing loss. The present study recruited 209 elderly individuals (all Chinese Han, ≥65 years, ≥8 years of education) including 116 aMCI subjects (mean ± SD age = 72.90±5.71 years, 70 men, 46 women, education = 14 years (range 8∼19 years)) and 93 healthy controls (mean ± SD age = 72.53±3.55 years, 44 men, 49 women, education = 14 years (range 11∼16 years)) from a memory clinic, five universities and some communities in Nanjing. They all gave informed consent to participate in this study, which was approved by the Institutional Ethical Review Board of the Clinic Medical College of Southeast University. Patients with aMCI and healthy controls did not differ significantly in gender or years of education (both P>0.05).

### Selecting candidate genes and SNPs, extracting genomic DNA and genotyping

Eighty-two SNPs were selected that were previously suggested as tagging SNPs based on the above hypotheses of AD, the Alzgene website and HapMap (in Table 1). For the genotyping of those gene polymorphisms, peripheral venous blood was withdrawn from each subject, and genomic DNA was extracted by using the TIANamp genomic DNA kit. Genotyping was performed by using the iPLEX Assay (SEQUENOM iPLEX® Gold Reagent Kit), which involved the assay design, DNA isolation, PCR amplification, SAP treatment, adjusting extension primers, iPLEX reaction, clean resin, dispensing to SpectroCHIP bioarray, and matrix-assisted laser desorption ionisation time-of-flight mass spectrometry (MALDI-TOF MS) analysis.

**Table 1:** Candidate genes and SNPs*.

hypothesis	candidate gene	SNP
cholinergic hypothesis	BChE-K	rs1803274
	AChE	rs2571598, rs3757869
	ChAT	rs3810950, rs2177369, rs1880676
amyloid cascade hypothesis	ACE	rs4343, rs1800764
	TOMM40	rs157581, rs2075650, rs157580, rs8106922
	APBB2	rs17443013
	BACE1	rs638405
	BACE2	rs28656880, rs9975138
	RTN3	rs10897445
	CR1	rs3818361
	CLU	rs2279590, rs11136000, rs9331888
	PICALM	rs3851179
	IL	rs1800587
	IL1B	rs1143627
	IL6	rs1800796
	TNFG	rs1799724, rs4645836
	ACT	rs4934
	IL10	rs1800896, rs1800871, rs1800872
	TGFB1	rs1800469
tau hypothesis	MAPT/STH	rs242557, rs2471738, rs242562
	GSK3β	rs334558, rs6438552, rs12630592
	LRP6	rs2302685, rs7316466, rs2284396, rs7294695, rs2417086
	CDK5	rs2069442
	CDC2	rs7919724, rs2448347
	DYRK	rs2835740, rs8126696
	CAMKII	rs2242255
cholesterol metabolism hypothesis	ApoE	rs429358, rs7412, rs769450, rs440446, rs405509
	LDLR	rs5925, rs11668477, rs12983082, rs2738444, rs1433099, rs688
	LRP1	rs1799986, rs2306692, rs1140648
	LRP8	rs3820198, rs3737983, rs5177
	ABCA1	rs2230806
	CH25H	rs4417181, rs17117126, rs7091822
	CYP1	rs754203, rs7157609, rs4900442
	SOAT1	rs2862616, rs3753526, rs1044925
	CST3	rs2424577, rs3827143
	MTHFR	rs1801133
	IDE	rs3758505,rs4646954
	PLAU	rs2227564

* All candidate genes and SNPs are based on four classic hypotheses: the cholinergic hypothesis, the amyloid cascade hypothesis, the tau hypothesis and the cholesterol metabolism hypothesis.

### Statistical analysis

Haploview version 4.0 was applied to analyse the Hardy-Weinberg equilibrium (HWE), minor allele frequency (MAF), the percentage of non-missing for each marker (%gene) and linkage disequilibrium and to delete the SNPs with a Hardy-Weinberg P value <0.001 or %gene <90 or minor allele frequency <0.05. Associations of alleles and genotypes with aMCI (by comparing allele, genotype and haplotype distributions between aMCI patients and normal controls) were analysed using Unphased version 3.3.13, and 1000 random permutations were performed with Unphased version 3.3.13 to correct P-values for multiple testing in the allelic, genotypic and haplotype association analyses. To investigate the influence of gene-gene interactions on the onset of aMCI, the generalised multifactor dimensionality reduction (GMDR) method was employed. Briefly, the n-dimensional space formed by a given set of SNPs is reduced to a single dimension to analyse n-way interactions, and score-based statistics using maximum-likelihood estimates are calculated to classify multifactor cells into two different groups (either high efficacy or low efficacy). All possible (two SNPs to five SNPs) interactions were further tested using 10-fold cross-validation in an exhaustive search that considered all possible variable combinations. The GMDR software provides a number of output parameters, including the cross-validation consistency, the testing balanced accuracy, and the empirical p-values, to assess each selected interaction. Permutation testing was used to provide empirical p-values of prediction accuracy as a benchmark based on 1000 shuffles. Missing data were imputed by the software mdrdt-0.4.3 before loading in GMDR. Other statistical analysis was performed using SPSS version 17.0. Interactions between two genes (one was ApoE ε4 and the other was any other candidate SNP) were performed using logistic regression analysis, and the differences between cases and controls were computed using the independent samples t-test for the normally distributed variables and the non-parametric Mann-Whitney U-test for the asymmetrically distributed variables. Significance levels were as follows for the two-tailed tests: P values <0.05 were regarded as significant.

## Results

### Neuropsychological evaluations

**Figure 1:**
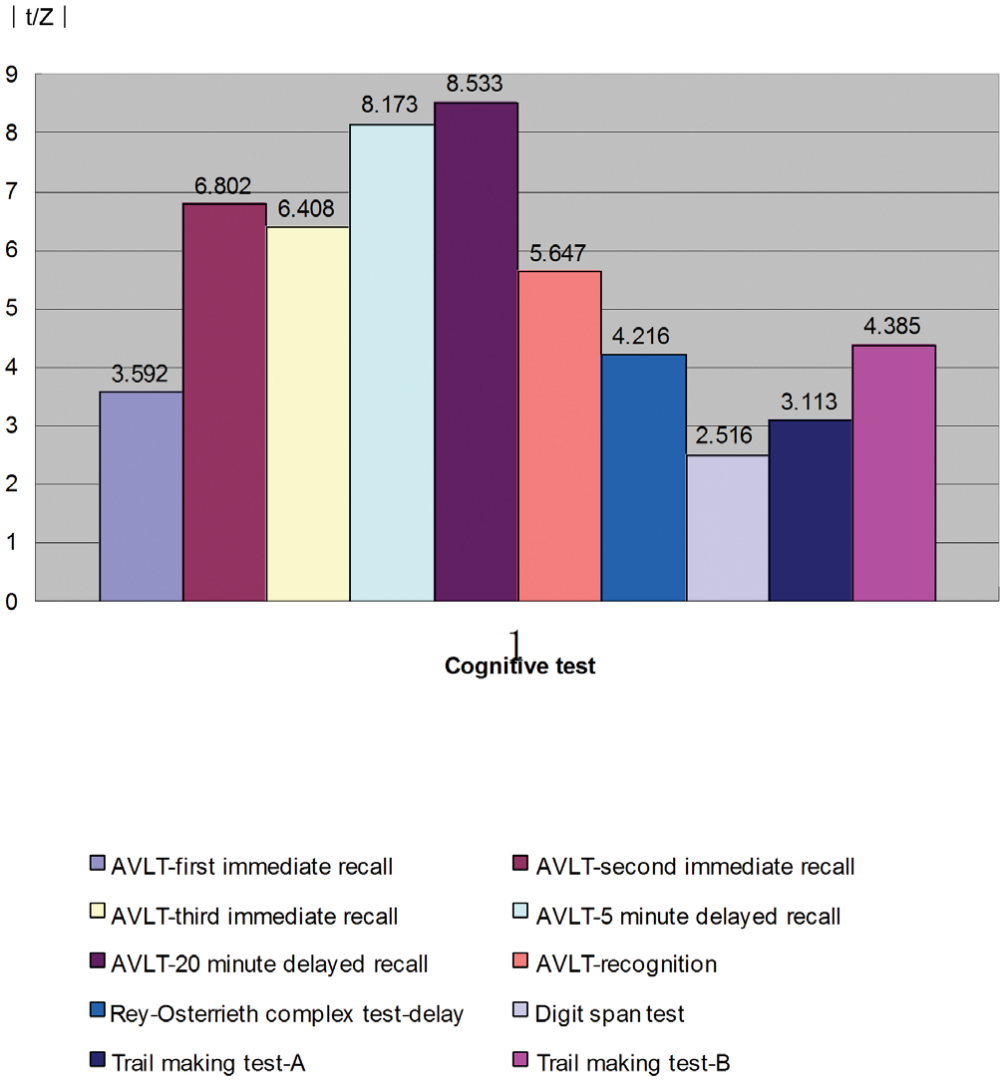
Comparison of cognitive performance between aMCI patients and normal controls. The scores from the neuropsychological tests (AVLT-first immediate recall, AVLT-second immediate recall, AVLT-third immediate recall, AVLT-5 minute delayed recall, AVLT-20 minute delayed recall, AVLT-recognition, Rey-Osterrieth complex test-delay, Digit span test, Trail making test-A, Trail making test-B) were significantly lower for the aMCI patients than for the normal controls (|t/Z| = 3.592, 6.802, 6.408, 8.173, 8.533, 5.647, 4.216, 2.516, 3.113, 4.385 respectively, all P<0.01).

The scores from the neuropsychological tests were significantly lower for the aMCI patients than for the normal controls (all P<0.01), with the largest impairments occurred on AVLT-20 minute delayed recall (Z = −8.533, P<0.001). Beyond the memory tasks, Trail Making Test B (representing executive function) showed the largest declines (t = −4.385, P<0.001), in Figure 1.

### SNP genotype and allele associations with aMCI

No SNP analysed had a Hardy-Weinberg P value <0.001, a %gene <90 or a MAF <0.05. The influence of the candidate SNP allele and genotype on the onset of aMCI was analysed, with the result that the distributions of SNPs relating to the amyloid cascade hypothesis (TOMM40 rs157581 G and TOMM40 rs2075650 G), to the cholesterol metabolism hypothesis (ApoE rs429358 C, LDLR rs11668477 G and CH25H rs7091822 T, PLAU rs2227564 CT) and to the tau hypothesis (MAPT/STH rs242562 GG) were significantly different than those in normal controls, the significant results are listed in Table 2. It was also confirmed that the distributions of ApoE ε4 haplotype in aMCI patients were significantly higher than those in normal controls (OR = 3.656, 95%CI = 1.267∼10.55, Χ^2^ = 14.13, P = 0.002). However, there were no differences in the genotypic or allelic distributions between aMCI patients and normal controls of SNPs relating to the cholinergic hypothesis (all P>0.05).

**Table 2:** Distributions of alleles and genotypes in candidate genes.

				Allele distribution		Genotype distribution			P value	
hypothesis	Gene	SNP	Sample	1	2	11	12	22	Allele	Genotype
amyloid cascade hypothesis	TOMM40	rs157581	Cases	168(0.7368)	60(0.2632)	62(0.5439)	44(0.3860)	8(0.0702)	0.024	0.071
		(A = 1, = 2)	Controls	155(0.8333)	31(0.1667)	63(0.6774)	29(0.3118)	1(0.0108)		
		rs2075650	Cases	198(0.8534)	34(0.1466)	86(0.7414)	26(0.2241)	4(0.0345)	0.013	0.028
		(A = 1, = 2)	Controls	174(0.9355)	12(0.0645)	81(0.8710)	12(0.1290)	0(0)		
tau hypothesis	MAPT/STH	rs242562	Cases	149(0.6478)	81(0.3522)	43(0.3739)	63(0.5478)	9(0.0783)	0.111	0.025
		(A = 1,G = 2)	Controls	105(0.5707)	79(0.4293)	32(0.3478)	41(0.4457)	19(0.2065)		
cholesterol metabolism hypothesis	ApoE	rs429358	Cases	36(0.1565)	194(0.8435)	4(0.03478)	28(0.2435)	83(0.7217)	0.001	0.001
		(C = 1, T = 2)	Controls	8(0.04348)	176(0.9565)	0(0)	8(0.08696)	84(0.9130)		
	LDLR	rs11668477	Cases	185(0.8110)	43(0.1890)	74(0.6490)	37(0.3250)	3(0.0260)	0.027	0.038
		(A = 1, = 2)	Controls	164(0.8910)	20(0.1090)	73(0.7930)	18(0.1960)	1(0.0110)		
	CH25H	rs7091822	Cases	28(0.1220)	202(0.8780)	0(0)	28(0.2430)	87(0.7570)	0.031	0.070
		(G = 1,T = 2)	Controls	37(0.2010)	147(0.7990)	3(0.0330)	31(0.3370)	58(0.6300)		
	PLAU	rs2227564	Cases	149(0.6480)	81(0.3520)	54(0.4700)	41(0.3560)	20(0.1740)	0.953	0.002
		(C = 1,T = 2)	Controls	120(0.6520)	64(0.3480)	33(0.3590)	54(0.5870)	5(0.0540)		

### Gene-gene interactions in aMCI

Gene-gene interactions were examined between two genes (one was ApoE ε4 and the other was any other candidate gene) and one significant result was obtained: the distributions of the DYRK1A rs8126696 CT genotype and the ApoE ε4 haplotype in aMCI patients were significantly lower than those in normal controls, as shown in Table 3 (OR = 0.100, P = 0.048). The high-order interactions for aMCI were first explored for the positive SNPs in Table 2, as a result, significant high-order interactions for aMCI were obtained and with covariable adjustments the best model which included LDLR rs11668477, PLAU rs2227564, and TOMM40 rs157581, scored 10 in the cross-validation consistency and 9 in the Sign Test (P = 0.011), as shown in Table 4. However, the reported positive SNPs were not possible to represent all the candidate SNPs from the four hypotheses together. High-order interactions were then explored for the SNPs in the four groups separately (the SNPs associated with the cholinergic hypothesis, amloid cascade hypothesis, tau hypothesis and cholesterol metabolism hypothesis). And significant high-order interactions for aMCI were also obtained from SNPs associated with the amloid cascade hypothesis but not from the SNPs merely associated with the cholinergic hypothesis, tau hypothesis or cholesterol metabolism hypothesis. With covariable adjustments, the best model which included TOMM40 rs157580 and BACE2 rs9975138, scored 10 in the cross-validation consistency and 10 in the Sign Test (P = 0.001), in Table 5.

**Table 3:** The interaction between DYRK1A rs8126696 and ApoE ε4 in aMCI.

locus	β	SE	OR	95% CI	P value
ApoE ε4	2.708	0.889	15.000	2.626–85.681	0.002
rs8126696 TT	-	-	-	-	0.014
rs8126696 CT	1.540	0.531	4.667	1.649–13.208	0.004
rs8126696 CC	1.141	0.483	3.130	1.215–8.063	0.018
ApoE ε4 * rs8126696 TT	-	-	-	-	0.140
ApoE ε4 * rs8126696 CT	−2.303	1.165	0.100	0.010–0.982	0.048
ApoE ε4 * rs8126696 CC	−1.210	1.113	0.298	0.034–2.639	0.277
Constant	−1.099	0.436	0.333	-	0.012

Abbreviations: SE: Standard Error; OR: odds ratio; CI: confidence interval.

**Table 4:** Comparison of best models, prediction accuracies, cross-validation consistencies and P values identified by GMDR in positive SNPs from Table 2 associated with aMCI.

Model	Testing Accuracy	cross-validation consistency	Sign Test(P)
PLAU rs2227564-TOMM40 rs157581	0.6081	8/10	9(0.011)
LDLR rs11668477-PLAU rs2227564-TOMM40 rs157581	0.6709	10/10	9(0.011)
LDLR rs11668477-PLAU rs2227564-TOMM40 rs157581-ApoE4	0.6173	5/10	8(0.055)
LDLR rs11668477-CH25H rs7091822-PLAU rs2227564- TOMM40 rs157581-MAPT\STH rs242557	0.6250	10/10	8(0.011)

**Table 5:** Comparison of best models, prediction accuracies, cross-validation consistencies and P values identified by GMDR in SNPs associated with amloid cascade hypothesis for aMCI.

Model	Testing Accuracy	cross-validation consistency	Sign Test(P)
TOMM40 rs157580-BACE2 rs9975138	0.6535	10/10	10(0.001)
IL1B rs1143627-TOMM40 rs157580-BACE2 rs9975138	0.5072	2/10	6(0.377)
IL1B rs1143627-ACT rs4934-TOMM40 rs157580-BACE2 rs9975138	0.6218	8/10	9(0.011)
IL1B rs1143627-ACT rs4934-TGFB1 rs1800469-BACE1 rs638405-PICALM rs3851179	0.4305	2/10	3(0.945)

## Discussion

In this study of genetic risk factors for aMCI, an important indicator of eventual development of AD, we have identified SNPs in single genes, and several interactions between these genes, that associate with this disorder.

The present study found that genes relating to the amyloid cascade hypothesis (TOMM40), tau hypothesis (MAPT/STH) and cholesterol metabolism hypothesis (ApoE, LDLR, CH25H, PLAU), but not the cholinergic hypothesis, influence susceptibility to aMCI. Moreover, the ApoE ε4 haplotype was associated with aMCI. Regarding gene-gene interactions in aMCI, using an association study based on those genetic hypotheses outlined previously, Interactions were considered to modulate the risk for aMCI in the Chinese population for the following: DYRK1A and ApoE ε4; LDLR, PLAU and TOMM40; and TOMM40 and BACE2.

Previous studies proposed that ApoE ε4 can not fully explain the association of 19q13 with AD risk and suggested that other functional variants near ApoE might be involved in modifying the effect of ApoE [12]. In the present study, TOMM40 rs157581 and rs2075650 but not TOMM40 rs8106922 showed an association with the onset of aMCI. This finding was consistent with the findings of other research groups. One group previously reported that the C allele of rs157581 was in very strong linkage disequilibrium with the C allele of rs429358 in ApoE and that TOMM40 might have less of an effect on the risk of LOAD in Caucasians [13]. Elsewhere, rs2075650, an intronic SNP of TOMM40, was reported to be associated with the Aβ42 level in normal subjects when ignoring age and the number of ApoE ε4 alleles [14]. The TOMM40 gene product is an essential transporter of proteins across the mitochondrial membrane.

MAPT/STH is mainly expressed in neurons and contributes to the organisation and integrity of the cytoskeleton. Previous studies found filamentous neuronal tau inclusions in many neurodegenerative diseases, including AD. However, an association between MAPT/STH rs242562 and AD/aMCI has not been verified, even though an association between Parkinson disease and a sub-haplotype involving SNP rs242562 received positive results [15]. In the present study, an association was found of MAPT/STH rs242562 in the aMCI that was attributable to the genotype GG.

It is well known that an elevated level of blood cholesterol can increase the risk of AD, although the exact mechanism remains unexplained. The present study found an association between LDLR rs11669576, CH25H rs7091822, PLAU rs2227564 and aMCI. The LDLR gene is located in 19p13, which has been reported to be associated with AD [16], and the LDLR protein can bind ApoE and transport cholesterol, thus having an effect on risk of AD. However, its ability to do so may vary between different genotypes [17]. Some researchers have identified a specific haplotype block of LDLR consisting of SNPs rs11669576, rs2738444 and rs5925 and showed the haplotype GTT was overrepresented in women affected with AD when compared to matched normal controls. It was also found that the haplotype GTT was associated with an increased level of tau and p-tau in both men and women independent of the ApoE allele [18]. Regarding CH25H, no report has addressed rs7091822. CH25H is capable of converting cholesterol to 25-hydroxycholesterol, and some studies have suggested that 25-hydroxycholesterol is a potent regulatory oxysterol, likely participating in several aspects of lipid metabolism. It was also suggested that CH25H played an important role in regulating gene expression and immune activation [19]. PLAU, located on chromosome 10q, was shown to be involved in the degradation of Aβ; however, studies on the association of PLAU rs2227564 (exon 6) and AD/aMCI found complex and discrepant results. Inconsistent with a finding from Riemenschneider's study with a higher frequency of the T allele in AD [20], one study of smaller sample size by Finckh et al. observed a lower frequency of the T allele in AD [21], and our study found a protective association with the CT genotype in aMCI.

ApoE ε4 has been found to be a genetic risk factor but not a deterministic factor for LOAD, and therefore, we attempted to find whether there is an interaction between other SNPs and ApoE in aMCI. As a result, our study found that the interaction between the DYRK1A rs8126696 CT genotype and the ApoE ε4 (+) haplotype was a protective factor for aMCI. The DYRK1A gene is located on human chromosome 21 and encodes a protein kinase that could phosphorylate or interact with several proteins such as the tau protein [22]. Kimura et al. investigated the DYRK1A gene and found an association with AD; they indicated that DYRK1A could be a key molecule bridging Aβ production and tau phosphorylation in AD [23].

As one single polymorphism or even one hypothesis is unlikely to unravel the mechanisms behind aMCI, the effect of gene-gene interactions on the onset of aMCI was further explored. Previous studies have not paid much attention to the interaction between LDLR, PLAU and TOMM40. Lamsa et al. did not find any association between LDLR rs11668477 and AD [18], but the present study could conclude that the results with LDLR, PLAU and TOMM40 indicate an interaction between cholesterol metabolism and Aβ production in aMCI. In addition, TOMM40 rs157580 and BACE2 rs9975138 were also involved in the metabolism of Aβ. Regarding TOMM40 rs157580, an Alzgene meta-analysis demonstrated a significant result for TOMM40 rs157580 G vs. A with an OR of 0.60. However, the present study of aMCI showed that aMCI did not significantly correlate with TOMM40 rs157580 variants, maybe due to the small sample size. The BACE2 gene is located on chromosome 21q22, and the accumulation of Aβ along with increased levels of BACE2 has been detected in patients with Down's syndrome [24]. However, it is quite controversial whether the BACE2 polymorphism is a genetic risk factor for AD. While some researchers suggested that BACE2 could cleave APP at the β-secretase site [25], others argued that BACE2 was not essential for the generation of Aβ [26]. The present study did not find an association between BACE2 and aMCI either, but found that TOMM40 rs157580 and BACE2 rs9975138 interactions could contribute to aMCI susceptibility, the first report of such a result in the Chinese population. Meanwhile, it was suggested that ApoE ε4 and BACE2 interactions were not associated with aMCI. Therefore, it is believed that TOMM40 variants may influence APP accumulation as a result of mitochondrial dysfunction and not just in linkage disequilibrium with ApoE [12], and the interactions between TOMM40 rs157580 and BACE2 rs9975138 may eventually increase the accumulation of Aβ.

In conclusion, this study has shown that aMCI is associated with SNPs in three systems relating to the pathogenesis of AD-those of the amyloid cascade, tau and cholesterol metabolism pathways. No association was observed with SNPs relating to the cholinergic hypothesis of AD. Interactions were also observed between genes in the amyloid pathway and between the amyloid and cholesterol pathways. In addition to providing clues as to the pathogenic mechanisms underlying the development of aMCI, these findings may contribute to establishing a profile of risk for AD in our population.

## References

[1] Petersen RC, Smith GE, Waring SC, Ivnik RJ, Tangalos EG (1999). Mild cognitive impairment: clinical characterization and outcome. Arch Neurol..

[2] Gauthier S, Reisberg B, Zaudig M, Petersen RC, Ritchie K (2006). International Psychogeriatric Association Expert Conference on mild cognitive impairment. Lancet..

[3] Perry EK (1986). The cholinergic hypothesis–ten years on. Br Med Bull..

[4] Glenner GG, Wong CW (1984). Alzheimer's disease: initial report of the purification and characterization of a novel cerebrovascular amyloid protein.. Biochem Biophys Res Commun.

[5] Wang JZ, Grundke-Iqbal I, Iqbal K (2007). Kinases and phosphatases and tau sites involved in Alzheimer neurofibrillary degeneration.. Eur J Neurosci.

[6] Benarroch EE (2008). Brain cholesterol metabolism and neurologic disease.. Neurology.

[7] Lehmann DJ, Johnston C, Smith AD (1997). Synergy between the genes for butyrylcholinesterase K variant and apolipoprotein E4 in late-onset confirmed Alzheimer's disease.. Hum Mol Genet.

[8] Ehehalt R, Keller P, Haass C, Thiele C, Simons K (2003). Amyloidogenic processing of the Alzheimer beta-amyloid precursor protein depends on lipid rafts.. J Cell Biol.

[9] Gotz J, Chen F, van Dorpe J, Nitsch RM (2001). Formation of neurofibrillary tangles in P301l tau transgenic mice induced by Abeta 42 fibrils.. Science.

[10] Rapoport M, Dawson HN, Binder LI, Vitek MP, Ferreira A (2002). Tau is essential to beta -amyloid-induced neurotoxicity.. Proc Natl Acad Sci U S A.

[11] Akiyama H, Barger S, Barnum S, Bradt B, Bauer J (2000). Inflammation and Alzheimer's disease.. Neurobiol Aging.

[12] Roses AD, Lutz MW, Amrine-Madsen H, Saunders AM, Crenshaw DG (2010). A TOMM40 variable-length polymorphism predicts the age of late-onset Alzheimer's disease.. Pharmacogenomics J.

[13] Yu CE, Seltman H, Peskind ER, Galloway N, Zhou PX (2007). Comprehensive analysis of APOE and selected proximate markers for late-onset Alzheimer's disease: patterns of linkage disequilibrium and disease/marker association.. Genomics.

[14] Han MR, Schellenberg GD, Wang LS (2010). Genome-wide association reveals genetic effects on human Abeta42 and tau protein levels in cerebrospinal fluids: a case control study.. BMC Neurol.

[15] Skipper L, Wilkes K, Toft M, Baker M, Lincoln S (2004). Linkage disequilibrium and association of MAPT H1 in Parkinson disease.. Am J Hum Genet.

[16] Wijsman EM, Daw EW, Yu CE, Payami H, Steinbart EJ (2004). Evidence for a novel late-onset Alzheimer disease locus on chromosome 19p13.2.. Am J Hum Genet.

[17] Kivipelto M, Helkala EL, Laakso MP, Hanninen T, Hallikainen M (2002). Apolipoprotein E epsilon4 allele, elevated midlife total cholesterol level, and high midlife systolic blood pressure are independent risk factors for late-life Alzheimer disease.. Ann Intern Med.

[18] Lamsa R, Helisalmi S, Herukka SK, Tapiola T, Pirttila T (2008). Genetic study evaluating LDLR polymorphisms and Alzheimer's disease.. Neurobiol Aging.

[19] Park K, Scott AL (2010). Cholesterol 25-hydroxylase production by dendritic cells and macrophages is regulated by type I interferons.. J Leukoc Biol.

[20] Riemenschneider M, Konta L, Friedrich P, Schwarz S, Taddei K (2006). A functional polymorphism within plasminogen activator urokinase (PLAU) is associated with Alzheimer's disease.. Hum Mol Genet.

[21] Finckh U, van Hadeln K, Muller-Thomsen T, Alberici A, Binetti G (2003). Association of late-onset Alzheimer disease with a genotype of PLAU, the gene encoding urokinase-type plasminogen activator on chromosome 10q22.2.. Neurogenetics.

[22] Song WJ, Sternberg LR, Kasten-Sportes C, Keuren ML, Chung SH (1996). Isolation of human and murine homologues of the Drosophila minibrain gene: human homologue maps to 21q22.2 in the Down syndrome “critical region”. Genomics.

[23] Kimura R, Kamino K, Yamamoto M, Nuripa A, Kida T (2007). The DYRK1A gene, encoded in chromosome 21 Down syndrome critical region, bridges between beta-amyloid production and tau phosphorylation in Alzheimer disease.. Hum Mol Genet.

[24] Acquati F, Accarino M, Nucci C, Fumagalli P, Jovine L (2000). The gene encoding DRAP (BACE2), a glycosylated transmembrane protein of the aspartic protease family, maps to the down critical region.. FEBS Lett.

[25] Farzan M, Schnitzler CE, Vasilieva N, Leung D, Choe H (2000). BACE2, a beta -secretase homolog, cleaves at the beta site and within the amyloid-beta region of the amyloid-beta precursor protein.. Proc Natl Acad Sci U S A.

[26] Cheon MS, Dierssen M, Kim SH, Lubec G (2008). Protein expression of BACE1, BACE2 and APP in Down syndrome brains.. Amino Acids.

